# Interaction between the Effects of Sustained Swimming Activity and Dietary Macronutrient Proportions on the Redox Status of Gilthead Sea Bream Juveniles (*Sparus aurata* L.)

**DOI:** 10.3390/antiox11020319

**Published:** 2022-02-06

**Authors:** Albert Sánchez-Moya, Miquel Perelló-Amorós, Emilio J. Vélez, Julia Viñuales, Isabel García-Pérez, Josefina Blasco, Joaquim Gutiérrez, Jaume Fernández-Borràs

**Affiliations:** Department of Cell Biology, Physiology, and Immunology, Faculty of Biology, University of Barcelona, 08028 Barcelona, Spain; alsanchezmo@ub.edu (A.S.-M.); miquelperelloamoros@gmail.com (M.P.-A.); emilio-jose.velez-velazquez@inrae.fr (E.J.V.); juliavinudal@gmail.com (J.V.); isabelgarcia@ub.edu (I.G.-P.); jblasco@ub.edu (J.B.); jgutierrez@ub.edu (J.G.)

**Keywords:** oxidative stress, high-lipid diet, high-protein diet, aerobic training, exercise, fish, red muscle, white muscle, liver, sea bream

## Abstract

The combination of physical exercise and a balanced diet presents substantial health benefits and could improve fish production. However, the redox balance can be affected by training regimen, dietary macronutrient ratio and their interaction. In this study, we conjointly evaluated the effects of physical activity (by voluntary swimming (VS) or sustained swimming as exercise (Ex)) and diet composition (by high-protein (HP) or high-lipid (HE) commercial diets) after 6 weeks on oxidative stress status in liver, white muscle and red muscle of gilthead sea bream juveniles. The HE diet increased the biochemical redox markers’ thiobarbituric acid reactive substances (TBARS), advanced oxidation protein products (AOPP) and reduced thiols (-SH) in the different tissues. Exercise increased AOPP and -SH levels in liver but reduced TBARS levels in white muscle. Regarding the expression of oxidative stress, chaperones and apoptosis-related genes, the VSHE group showed the highest values and the VSHP the lowest, whereas the application of sustained swimming partially equalized those differences. Diet composition modulated the enzyme activity, prioritizing the superoxide dismutase and catalase in the HE-fed groups and the glutathione-related enzymes in the HP groups. Exercise also altered enzyme activity, but in a tissue-dependent manner. Overall, the redox balance in gilthead sea bream juveniles can be affected by diet composition and sustained swimming. However, the response will partly depend on the interaction between these factors and the tissue studied. Therefore, the combination of an adequate diet and sustained exercise could be used in fish production to improve the physiological redox status.

## 1. Introduction

The increasing demand for healthy, safe and sustainable food supplies with the growth of the human population has become a worldwide challenge that can be partly addressed by aquaculture products, which currently account for more than 50% of the global fish consumption. Fish feeding represents about 50% of the total fish production costs, with fish meal and fish oil being the most valued raw materials. However, these nutrient sources need to be replaced by more sustainable alternatives and practices without compromising fish growth, welfare, product quality and competitiveness. The most valued cultivated fish are carnivorous species that have a high dietary protein requirement (40~60%), used as the preferred energy and anabolic substrates. However, the global trend is to reduce the use of proteins in favor of cheaper ingredients such as lipids or carbohydrates [1]. Lipids are an excellent energy source that spare proteins from catabolism and improve muscle-gaining efficiency. However, their inclusion in feed is physiologically limited as their excessive consumption could lead to metabolic impairment, resulting in a pathological condition [2,3,4,5]. Increasing the levels of dietary energy proceeding from lipids is associated with a higher fat deposition, particularly in the liver, adipose tissue and muscle. It is well established that raising the dietary lipid content leads to the production of reactive oxygen species (ROS) through diverse pathways, generating downstream molecules such as malondialdehyde (MDA), advanced oxidation protein products (AOPP) and oxidized thiols, which can be used as biochemical markers of the tissue redox status in fish [6,7]. Marine fish feed is rich in polyunsaturated fatty acids (PUFAs), which accumulate in tissues and provide a healthy meal for human consumption. However, their chemical structure makes them more susceptible to peroxidation, enhancing the prooxidant state [4,8]. At the same time, the level of lipids in feed modulates the activity of redox enzymes such as superoxide dismutase (SOD) and glutathione-related ones [9,10,11].

Gilthead sea bream (*Sparus aurata*), the most important marine fish currently cultivated in the Mediterranean area, naturally carries out sustained swimming during the day [12]. However, this natural activity is lowered by tank-rearing methods and domestication, which prioritizes growth rate at the detriment of aerobic capacities [13]. The positive effects of physical activity on fish performance have been previously demonstrated in some species. For example, exercise improves hormonal regulation, growth, muscle remodeling and flesh quality [14,15,16,17,18,19], metabolism and the use of nutrients [20,21], stress mitigation, the immune status and neuroplasticity, supporting its potential application in fish farming [22,23]. Nonetheless, other similar studies have not found differences between exercising and resting fish or have even reported negative effects of exercise such as a reduction in growth and feed efficiency as well as increases in oxidative stress. This illustrates the complex physiological response to exercise and the relevance of redox analytics [24,25,26,27]. Exercise generates a systemic effect and promotes numerous biochemical and enzymatic adaptations, producing a major impact on the liver and skeletal muscle. Depending on the growth stage, muscle represents 30 to 60% of the body weight (BW) in sea bream, playing a key role in energy and redox homeostasis [28,29]. Thus, fish are an interesting experimental model given their anatomically separated muscle fiber types. Fish have a predominant type IIB or fast glycolytic white muscle fiber (~30–50% BW) and type I or slow oxidative red muscle fiber (~4–10% BW), each presenting specific functionality in relation to contractile activity [30,31]. Overall, the response of fish to moderate sustained swimming is qualitatively similar to that of mammals performing endurance exercise, turning into a more aerobic phenotype, inducing mitochondrial biogenesis and angiogenesis, as well as modulating the redox balance, which has been rarely investigated [14,15,17,18,26,27,32]. In mammals, it is well established that increased physical activity enhances the production of ROS through different pathways, including several oxidases and electron leak during mitochondrial respiration (0.15–3% of the total flow) [33,34,35,36]. At moderate and physiological concentrations, ROS improve force generation and are essential upstream signals for multiple redox-sensitive transcription factors, including self-protective redox defenses. However, if the antioxidant-buffering capacity of the cell is exceeded, the integrity of several cellular structures and molecules is compromised, leading to pathologies and aging. Hence, considering culture conditions such as the diet composition and temperature and based on the hormesis theory, exercise could be considered a friend or foe depending on the training regimen in terms of intensity and duration [27,36]. The synergic benefits of regular physical activity and a balanced diet have long been acknowledged in mammals, focusing on health and lifespan. However, the combination of these approaches as a tool to promote productivity and welfare in fish farming from the point of view of oxidative stress is still to be explored in detail.

The sedentary lifestyle of fish in traditional tank-based farming alongside a high-lipid diet could lead to suboptimal productivity. Sustained aerobic exercise is a feasible application to improve the use of nutrients as fuels. Hence, the training regimen and its interactions with diet composition must be determined for each species and size, since an imbalance could lead to an excessive prooxidant condition. Accordingly, in the present study, we investigated the effect of the diet composition (a high-protein vs. a high-lipid diet) and exercise regimen (voluntary swimming vs. moderate sustained aerobic swimming) on oxidative stress in the liver and the white and red muscles of gilthead sea bream juveniles after 6 weeks of the trial.

## 2. Materials and Methods

### 2.1. Animals and Experimental Conditions

Gilthead sea bream fingerlings (4.1 ± 0.1 g mean body weight) obtained from a local fish farmer (Piscimar, Burriana, Spain) were maintained in the facilities of the Faculty of Biology (University of Barcelona) at a constant temperature of 23 ± 1 °C, 38‰ salinity and under a 14L/10D photoperiod in a semi-closed recirculating system. After one week of acclimation, the fish were distributed randomly into eight 200 L and two 400 L fiberglass tanks at the same biomass density (1.5 kg/m^3^). The voluntary swimming (VS) group was placed in the 400 L tanks under a vertical water inflow, where the fish swam at will. The sustained swimming group (exercised, Ex) was placed in the 200 L tanks, where a circular laminar flow was generated by tangentially directing the water inflow through a plastic flute. The flow of each tank was regulated to achieve an initial speed of 2.5 body lengths (BL)/s, which represents an adequate sustained aerobic training regimen previously described for this species [15,29]. The flow speed decreased proportionally to the increase in fish size, with a 20% reduction at the end of the trial, as previously reported by Moya et al. (2019) [17].

Two commercial diets were obtained from Skretting España S.A. (Burgos, Spain), differing mainly in the macronutrient ratio (Table 1). One was a high-protein diet (HP, 54% protein/15% lipid) and the other a high-lipid diet (HE, 50% protein/20% lipid). Both diets were formulated with the same ingredients: fishmeal, fish oil, cereal products and byproducts (wheat gluten, corn gluten and wheat flour), legume products and byproducts (soybean meal and pea starch), vitamins and minerals. Both diets contained a high enough quantity of the main PUFA to cover the minimum fish requirements. Half of the tanks (one of the 400 L and four 200 L tanks) were fed the HP diet, while the other half were fed the HE diet. The daily ration was set at 5% of the total biomass for each tank distributed in 3 meals. The pellet size was increased from 1.5 mm to 1.9 mm three weeks after the beginning of the trial.

### 2.2. Sampling

After six weeks of the experimental trial, the fish were fasted overnight before being sacrificed by an anesthesia overdose (300 mg/L ethyl 3-aminobenzoate methanesulfonate, MS-222, Sigma^®^, Madrid, Spain). Death was confirmed by sectioning the spinal cord. Biometric data and somatic indices were recorded for all the fish in Perelló-Amorós et al. (2021) [18]. Twelve fish from each voluntary swimming tank (*n* = 12 for VSHP and VSHE groups) and sixteen fish per condition from exercised tanks, four per tank (*n* = 16 for ExHP and ExHE groups), were sampled. Samples of liver as well as skeletal red and white muscle were frozen immediately in liquid nitrogen and stored at −80 °C until further analysis.

### 2.3. Biochemical Markers and Enzyme Activity

Liver, red muscle and white muscle pieces were homogenized with 1:9 (*w*/*v*) cold buffer (100 mM Tris-HCl, 1 mM EDTA and 0.1% Triton X-100 (*v*:*v*), pH 7.6) in a Precellys^®^ Evolution homogenizer (Bertin Technologies SAS, Montigny-le-Bretonneux, France) at 4 °C, following the manufacturer’s recommendations. Briefly, 50–100 mg of fresh tissue was placed in 2 mL Precellys^®^ tubes with buffer and zirconium beads (1.4 and 2.8 mm) and homogenized with four to six short pulses (10 to 20 s) at different intensities (5500–6500 rpm), depending on the structure of the tissue. A first centrifuge was performed at 2500× *g* for 5 min to remove the debris, aliquoting part of the supernatant for MDA-TBARS and AOPP analysis. A second centrifuge was performed for 20 min at 16,000× *g* for the liver samples and at 13,000× *g* for the red and white muscle homogenates, discarding the pellet and aliquoting the supernatant for the remaining biochemical and enzymatic analyses.

Soluble protein concentrations (mg) used for the normalization of the biochemical markers and enzyme activity were determined with the Bradford method [37].

All biochemical markers were analyzed in the liver and white muscle samples. Due to the scarce amount of available red muscle samples, only TBARS was analyzed in this tissue. Lipid peroxidation was evaluated using the thiobarbituric acid reactive species (TBARS) assay, following the procedure of Mihara and Uchiyama (1978) [38] with some modifications [39]. TBA reacts with the end reactive product of lipid peroxidation malondialdehyde (MDA). The resulting TBA-MDA adduct was fluorometrically measured (λ_ex_ = 515 nm; λ_em_ = 548 nm). Malondialdehyde tetrabutylammonium salt (Sigma, Madrid, Spain) was used for the standard curve. Lipid peroxidation is expressed as μmol of the MDA adduct per mg of protein. Advanced oxidation protein product (AOPP) levels were assayed using a modified method of Witko-Sarsat et al. (2003) [40] that had been adapted for fish by Sánchez-Nuño et al. (2019) [39]. Briefly, AOPP formation was measured at 340 nm using a standard calibration curve of 200 µL of chloramine-T solution (0 to 200 µM), 10 μL of 1.16 M potassium iodide, and 20 μL of acetic acid. Thus, the AOPP concentration is expressed as μM of chloramine-T equivalents per mg of protein. Concentrations of the total sulfhydryl groups (protein and non-protein thiols, -SH) were determined by the Sedlak and Lindsay method (1968) [41]. Ellman’s reagent (also called 5,5’-dithiobis-(2-nitrobenzoic acid) or DTNB) reacts with a free sulfhydryl group to yield mixed disulfide and 2-nitro-5-thiobenzoic acid (TNB^−^), which has a maximum adsorption at 412 nm. Reduced glutathione was used as the standard, and the results are expressed as µmol of sulfhydryl group per mg of protein.

Superoxide dismutase (SOD, EC 1.15.1.1.) activity was analyzed using a commercial kit (SOD Activity Assay kit KB03011, Bioquochem, Gijon, Spain), according to the manufacturer’s instructions. This kit utilizes a tetrazolium salt to detect the superoxide radicals generated by xanthine oxidase and hypoxanthine. The superoxide anions reduce the tetrazolium salt into a colored formazan product that has a maximum absorption at 450 nm. SOD scavenges superoxide anions, thereby reducing the rate of formazan dye formation. Catalase (CAT, EC 1.11.1.6) activity was assayed following the method of Aebi (1984) [42] with minor modifications. The decreasing optical density of hydrogen peroxide (H_2_O_2_) was registered at 240 nm. A 50 mM potassium phosphate buffer (pH 7.0) and freshly prepared 10.6 mM H_2_O_2_ were used as the reaction media for measuring CAT activity. Glutathione S-transferase (GST, EC 2.5.1.18) activity was analyzed following the method of Mannervik (1985) [43]. GST conjugates 1-chloro-2,4-dinitrobenzene (CDNB) to reduced glutathione (GSH). The increasing absorbance at 340 nm was registered. Glutathione peroxidase (GPX, EC 1.11.1.9) activity was assessed by measuring the consumption of NADPH at 340 nm [44]. Measurements were performed in 50 mM potassium phosphate buffer (pH 7.2) containing 1 mM EDTA, 2 mM sodium azide, 0.5–1 U/mL of glutathione reductase, 2 mM reduced glutathione and 0.1 mM NADPH. Glutathione reductase (GR, EC 1.8.1.7) activity was analyzed by measuring NADPH oxidation at 340 nm [45]. Assays were performed in 0.1 M potassium phosphate buffer (pH 7.5) containing 1 mM EDTA, 0.63 mM NADPH and 3.25 mM oxidized glutathione.

### 2.4. RNA Extraction, cDNA Synthesis and Real-Time Quantitative PCR (qPCR)

Total RNA was extracted from 40 mg of liver or 100 mg of white muscle or red muscle. These were homogenized in 1 mL of TRI Reagent^®^ using the Precellys^®^ Evolution homogenizer (Bertin-Corp, Rockville, MD, USA) and cooled at 4–8 °C. After homogenization, RNA extraction was performed following the manufacturer’s protocol. A NanoDrop 2000 spectrophotometer (Thermo Scientific, Alcobendas, Spain) was used to determine RNA concentration and purity. An RNA integrity check was performed with 1% agarose gel stained with 3% SYBR Safe DNA gel stain (ThermoScientific, Alcobendas, Spain). Then, 1 µg of RNA was treated with DNase I (Life Technologies, Alcobendas, Spain) following the manufacturer’s recommendations to eliminate any residual genomic DNA before cDNA synthesis. Finally, reverse transcription was performed with the Transcriptor First Strand cDNA Synthesis Kit (Roche, Sant Cugat del Vallès, Spain), using anchored oligo(dT)15 and random hexamer primers.

Gene expression (mRNA) analyses were performed via qPCR with the cDNA samples, according to the guidelines of MIQUE [46] in Hard-Shell^®^ 384-well PCR plates and a CFX384TM Real-Time System (Bio-Rad, El Prat de Llobregat, Spain). The analyses were performed in triplicate using 2.5 μL of the iTaq Universal SYBR Green Supermix (Bio-Rad, El Prat de Llobregat, Spain), 250 nM of the forward and reverse primers and 1 μL of diluted cDNA for each sample made up to a final volume of 5 μL. The reactions consisted of an initial denaturation step of 3 min at 95 °C, 40 cycles of 10 s at 95 °C, 30 s at 60–69 °C (primer dependent) followed by an amplicon dissociation analysis from 55 to 95 °C at a 0.5 °C increase every 30 s. The sequences, melting temperatures and accession numbers of the primers used in the Real-Time quantitative PCR analysis are displayed in Supplementary Table S1. For the relative expression calculations, different reference genes were used depending on the tissue: elongation factor 1 *alpha* (*ef1α*), ribosomal protein S18 (*rps18*), *beta*-actin (*β-actin*) and ribosomal protein L27 (*rpl27*), according to their stability, which was confirmed with the geNorm algorithm implemented in the Bio-Rad CFX Manager 3.1 software. The relative gene expression was standardized for each group (VSHP, VSHE, ExHP and ExHE) against the mean expression of each gene, followed by a standard score normalization (log2). These data are presented as heat maps for the liver, red muscle and white muscle samples, with the purple boxes showing the downregulation of the relative expression and the yellow ones indicating upregulation.

### 2.5. Statistics

Data were analyzed using the IBM SPSS Statistics version 22 software and plotted as mean + SEM with GraphPad Prism version 7 (GraphPad Software, La Jolla, CA, USA). Data were tested for normality with the Shapiro–Wilk test and for homogeneity of variances with Levene’s test. The differences between the four experimental groups were analyzed via two-way ANOVA, with diet (D; HE, HP), exercise (E; VS, Ex) and their interaction (I; D × E) as independent factors. Interaction means that using a factor (e.g., diet) as a reference, the other factor (exercise) acts different for the HP and HE groups. Significant differences are expressed as: -, not significant; * *p* ≤ 0.05; ** *p* ≤ 0.01 and *** *p* ≤ 0.001. The N of the groups were: VSHP = 12; VSHE = 12; ExHP = 16 and ExHE = 16.

Data for the background heat map were standardized against the mean value of each biochemical marker in each tissue. Purple and yellow indicate the lowest and highest concentration levels, respectively, whereas white represents the 50th percentile.

## 3. Results

### 3.1. Redox Metabolites

The concentrations of the oxidative-stress-related biomarkers for lipid (TBARS, µmol MDA/mg protein) and protein peroxidation (AOPP, µEq Ch-T/mg protein) and the level of total reduced thiols (-SH, µmol/mg protein) in the liver, white muscle and red muscle are shown in Table 2.

In the liver, the HE diet (VSHE and ExHE), compared to the HP diet (VSHP and ExHP), significantly increased the levels of TBARS (52% vs. 32.4%), AOPP (51.5% vs. 22.2%) and -SH (40.6% vs. 14.4%). Furthermore, compared to voluntary swimming (VSHP and VSHE), sustained swimming (ExHP and ExHE) increased AOPP (39.8% vs. 22.2%) and -SH (28.3% vs. 4.5%) levels, but not the TBARS level. The interaction (I) between diet and exercise did not show differences.

White muscle also presented increased levels of TBARS (32.6% vs. 16.2%) and AOPP (78.6% vs. 30.8%) in fish on the HE diet compared to those on the HP diet. The TBARS (−24.2% vs. −24.6%) level was reduced in fish performing sustained swimming (ExHP and ExHE), but not the AOPP level. Neither diet nor exercise affected total -SH levels in this tissue.

In the red muscle, as in the liver and white muscle, the HE diet also raised the TBARS level (302.5% vs. 139.4%) compared to the HP diet. Exercise and the interaction between diet and exercise had no effect in this tissue.

### 3.2. Relative Gene Expression

**Figure 1:**
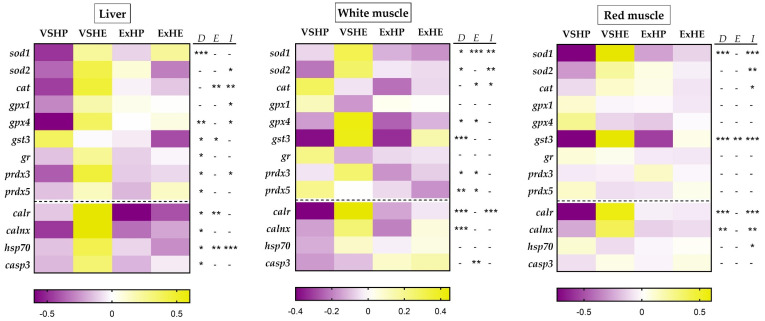
Comparative heat maps showing the changes in the expression of the genes associated with oxidative stress as well as those encoding chaperones and proapoptotic marker in the liver (left), white muscle (center) and red muscle (right) of gilthead sea bream fed a high-protein (HP) or high-energy (HE) diet and performing voluntary swimming (VS) or sustained swimming (Ex) for 6 weeks. Gene expression was first calculated relative to the corresponding reference genes for each tissue. It was then standardized following a standard score normalization (log2) against the mean value of each gene in each tissue. Shades of purple and yellow indicate the lowest and highest expression levels, respectively, as specified in the scale bar of the figure. Two-way ANOVA with diet (D), exercise (E) and their interaction (I = D x E) as independent factors was performed for each gene. -, not significant; * *p* ≤ 0.05; ** *p* ≤ 0.01; and *** *p* ≤ 0.001.

The relative expressions of the genes associated with oxidative stress and those encoding chaperones and proapoptotic markers in the liver, white muscle and red muscle are shown in Figure 1 as heat maps. A similar pattern was found in the three tissues for the two different diets, but was more marked in the liver, where the HE diet (VSHE group) consistently upregulated (yellow) the majority of the genes compared to the HP diet (VSHP group), which produced the lowest values (purple). However, when fish were subjected to sustained swimming (ExHP and ExHE groups), the differences between the dietary groups diminished. In the liver, diet composition had more significant effects (seen in the expression of *sod1*, *gpx4*, *gst3*, *gr*, *prdx3*, *prdx5*, *calr*, *calnx*, *hsp70* and *casp3*) compared to exercise (*cat*, *gst3*, *calr* and *hsp70*). Furthermore, an interaction between the effects of the diet composition and exercise was observed for *sod2*, *cat*, *gpx1*, *gpx4*, *prdx3* and *hsp70*. Interestingly, only *gst3* showed an opposite effect, with higher values for the HP groups and a global reduction in fish performing sustained swimming.

In the white muscle, the gene expression pattern was similar to that of the liver, but with some differences. Generally, the highest expression values were observed for the VSHE group (*sod1*, *sod2*, *gpx4*, *gst3*, *prdx3*, *calr* and *calnx*), but three genes (*cat*, *prdx5* and *casp3*) had a different expression pattern. Even so, the lowest values were mostly found in the groups fed the HP diet (VSHP and ExHP). The effect of diet was found in the expression of *sod1*, *sod2*, *gpx4*, *gst3*, *prdx3*, *prdx5*, *calr* and *calnx*, while the effect of exercise was observed for *sod1*, *car*, *gpx4*, *prdx3*, *prdx5* and *casp3*. An interaction between the effects of diet and exercise was found for *sod1*, *sod2*, *cat* and *calr*. *gst3*, and *calr* showed an acute color contrast among the groups on the heat maps, especially those performing voluntary swimming, suggesting a precise genetic regulation of these enzymes in this tissue.

In the red muscle, the effect of diet was observed for *sod1*, *gst3*, *calr* and *calnx*, whereas the effect of exercise was only noted for *gst3*. An interaction between the effects of diet and exercise was observed for *sod1*, *sod2*, *cat*, *gst3*, *calr*, *calnx* and *hsp70*. *Sod1*, *gst3* and *calr* presented the most intense color contrast among the groups on the heat maps, reinforcing the idea that these proteins have an important role in regulating the muscle redox status. Even so, the genes that showed significant differences presented a similar pattern to that found in the liver, except for *gst3*. No differences were observed for the genes encoding glutathione-related enzymes (*gpx1*, *gpx4*, and *gr*) and peroxiredoxins (*prdx3* and *prdx5*).

### 3.3. Enzyme Activity

**Figure 2:**
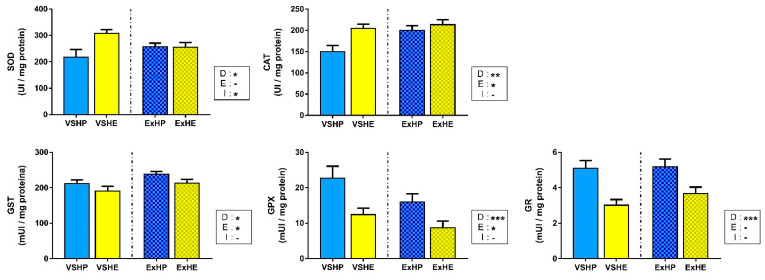
Activities of superoxide dismutase (SOD), catalase (CAT), glutathione S-transferase (GST), glutathione peroxidase (GPX) and glutathione reductase (GR) in the liver of gilthead sea bream fed a high-protein (HP) or high-lipid (HE) diet and performing voluntary swimming (VS) or sustained swimming (Ex) for 6 weeks. Data are expressed as the mean ± SEM. Two-way ANOVA with diet (D), exercise (E) and their interaction (I = D x E) as independent factors was performed for each enzyme: -, not significant; * *p* ≤ 0.05; ** *p* ≤ 0.01; and *** *p* ≤ 0.001.

The activities of antioxidant enzymes in the liver in response to the different diets and exercise are shown in Figure 2. SOD and CAT showed significantly increased activities in the VSHE group. However, the differences were equalized when fish were subjected to sustained swimming, being reduced for SOD activity and increased for CAT activity. On the other hand, the enzymes associated with glutathione (GST, GPX and GR) showed the opposite pattern, with a clear reduction in the activity of fish fed the HE diet compared to those on the HP diet. Sustained swimming also affected enzyme activities, increasing GST activity and decreasing GPX activity.

**Figure 3:**
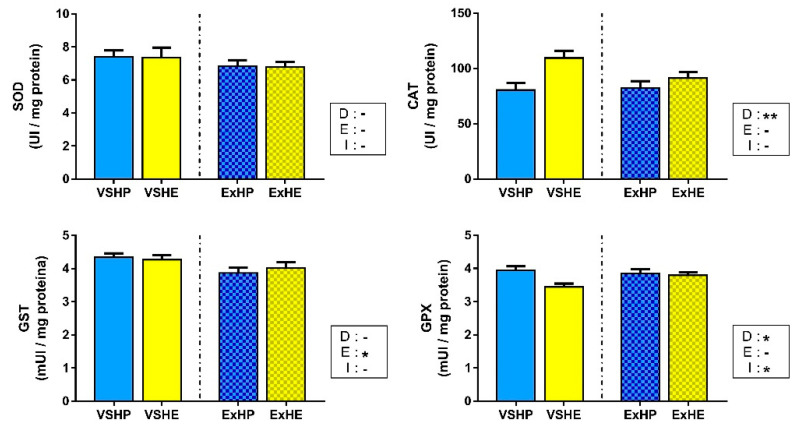
Activities of superoxide dismutase (SOD), catalase (CAT), glutathione S-transferase (GST) and glutathione peroxidase (GPX) in the white muscle of gilthead sea bream fed a high-protein (HP) or high-energy (HE) diet and performing voluntary swimming (VS) or sustained swimming (Ex) for 6 weeks. Data are expressed as the mean ± SEM. Two-way ANOVA with diet (D), exercise (E) and their interaction (I = D x E) as independent factors was performed for each enzyme: -, not significant; * *p* ≤ 0.05; ** *p* ≤ 0.01.

Enzyme activities in the white muscle are shown in Figure 3. SOD activity did not show differences among the groups, while CAT activity was only increased in fish fed the HE diet. No effect of exercise was found for these enzymes. GST activity decreased when fish performed sustained swimming, but no differences were found between the different diets. GPX activity was reduced in both HE groups (VSHE and ExHE), but was not affected by the swimming condition. However, an interaction between these factors was found, reducing the differences between diets when sustained swimming was applied. GR activity was below the limits of detection in the white muscle; hence, this enzyme is not included in Figure 3.

## 4. Discussion

The consequences of diet composition and the exercise regimen have been widely studied separately in vertebrates. However, the combined effects of these two factors on the redox status are less known. Mammals and fish share the main protective mechanisms against oxidant molecules, involving enzymes (SOD, CAT, GPX, GST, GR and PRDX), antioxidant compounds (glutathione, vitamins, selenium, etc.) and NADPH as the main reducing power. However, fish produce proportionally higher rates of ROS, have lower activities of antioxidant enzymes and present more PUFA in cell membranes, which boost lipid peroxidation [2,8]. Therefore, the evaluation of the redox balance is relevant to establish the physiological status of fish.

The present work is the first to study the combined effect of diet (two commercial diets differing in their lipid/protein ratios) and exercise (voluntary or sustained swimming) on oxidative stress in the liver, red muscle and white muscle of gilthead sea bream after 6 weeks of the trial.

### 4.1. Effect of Diet on Oxidative Stress Status

The two commercial diets tested in this study differed in their energy (HP, 18 MJ/kg; HE, 19.9 MJ/kg), lipid (HP, 15%; HE, 20%) and protein (HP, 54%; HE, 50%) contents. Two main factors explain our results regarding diet. First, as previously mentioned, increasing the levels of dietary lipid and energy is associated with a higher systemic fat deposition, which, alongside the high level of PUFA in fish feed and in their own tissues, enhances lipid peroxidation. Second, dietary protein could affect the oxidative stress balance, as this macronutrient can supply the sulfur amino acids required for GSH synthesis. Furthermore, the carbon skeleton of proteins can be used for gluconeogenesis, during which glucose directly scavenges ROS and provides reducing power through the pentose phosphate pathway in the form of NADPH [47,48,49].

A higher lipid or protein intake would increase their tissue deposition, as well as their use as energy substrates and in peroxidation [2,4]. However, our results showed that the biochemical peroxidation markers associated with lipids (TBARS) and proteins (AOPP) were consistently elevated in all the tissues by the HE diet, but not the HP diet. This was observed in all the tissues but was particularly pronounced in the red muscle and liver of fish fed the HE diet, with increases from 32.4% to 302.5%. The lipid storage function of the liver and the preference of red muscle to use fatty acids as fuel explain these tissue-dependent increases [31,32]. These results agree with those of studies showing that a higher lipid content in feed increases both lipid and protein peroxidation markers from 10 to 90% [4,6,11]. Interestingly, the reduced AOPP levels in the HP groups could be correlated with the lower nitrogen fractionation (Δδ^15^N) found in the white muscle of the same fish analyzed in this work [18]. This decrease in nitrogen fractionation indicates that the amino acids from dietary proteins need to be less metabolically interconverted before being deposited in muscle proteins. Therefore, the higher values of δ^15^N in the muscle of the groups fed the HE diet would be a consequence of a greater use of proteins and, consequently, more peroxidated. The higher levels of TBARS and AOPP indicated a prooxidant condition that, in the long term, could lead to cellular damage and several pathologies in the HE groups. In addition, from the point of view of fish farming, it would reduce fillet gain, quality and shelf life [50]. Despite an enhanced prooxidant environment in the HE groups, the increased levels of hepatic reduced thiols (-SH) suggested that a harmful condition had not yet been reached. Karaman et al. (2013) [6] found an increase in -SH levels in the blood and liver of rats fed a high-fat diet after 90 days of the trial, probably to buffer the increase in the oxidant byproducts. Nevertheless, they also found a significant reduction after 180 days, suggesting a first compensatory process that is exceeded and depressed after a while. There were no significant interactions between the effects of diet and exercise on the biochemical biomarkers. However, the differences in the effects of the two diets were slightly reduced by exercise, as previously observed by Zacarias et al. (2017) [51] in the rat liver and by Camiletti-Moirón et al. (2014) [52] in the brain.

The transcriptional regulation of oxidative-stress-related genes and enzyme activity were also highly affected by the diet composition in the different tissues. Liver showed the most significant differences, which was expected given its key role in metabolic homeostasis and detoxification by redox reactions. Liver showed a consistent upregulation of the main oxidative-stress-related protection mechanisms in the VSHE group compared to the VSHP group, with these differences generally reduced by exercise (ExHE vs. ExHP). One of the first defenses against oxidative stress involves SOD, which catalyzes the conversion of superoxide into hydrogen peroxide in the cytosol (Cu/Zn SOD or SOD1) and mitochondrial matrix (MnSOD or SOD2), and whose expression and activity can be modulated by the diet composition [4,53,54]. In our study, *sod1* and *sod2* showed the same pattern of expression in all the tissues for both diets, but with more marked differences for *sod1*, suggesting a slightly higher effect of lipid levels on the cytosol than the mitochondria. A few studies are available on the modulation of the relative gene expression of *sod* isozymes by diet or exercise, with the predominant techniques used being Western blots and enzyme activity analysis. In accordance with our results, Takahashi et al. (2002) [55] found an increase in *sod2* expression in mouse liver when the dietary lipid content was increased. Additionally, Pérez-Jiménez et al. (2009) [4] showed a different protein expression of SOD1 and SOD2 in *Dentex dentex* depending on the tissue and the amount of dietary protein and lipid contents. Our observation of increased mRNA levels of *sod1* and *sod2* in the liver of the VSHE group correlated with a higher total SOD activity, in accordance with results found in other species [3,4,9]. However, no differences in SOD activity were found in the white muscle, suggesting a different post-transcriptional regulation. The hydrogen peroxide produced by SOD is converted into water by CAT, which is mainly located in peroxisomes and the mitochondria [56]. No significant differences in *cat* expression were found for the two diets, although the heat map color for *cat* was similar to that for *sod* except in the white muscle. The increase in CAT activity in the liver and white muscle of the HE groups demonstrates its importance in the regulation of hydrogen peroxide, which is in agreement with previous studies reporting increases in CAT expression and activity in fish and mammals following an increased dietary lipid intake [3,4,9,10]. However, the opposite results were shown by Lasker et al. (2019) [11], who observed that a high-fat diet reduced the activities of SOD and CAT. This is in line with the results observed in murine models by Karaman et al. (2013) [6] for reduced thiols and by Jarukamjorn et al. (2016) [57] for relative expression and enzyme activity, with an initial upregulation of protective mechanisms that is exceeded and depressed after a while.

Hydrogen peroxide and other small organic hydroperoxides act as pro-oxidant compounds and are very important signaling molecules [58]. These compounds can also be reduced by complementary mechanisms such as the GSH-dependent pathway through the action of the GPX-GR-GST and PRDX-TRX systems. All of them depend directly on the availability of the reducing power of NADPH, which is higher in animals fed diets that are rich in carbohydrates or proteins rather than lipids [45,48,59,60]. GPX has different isoforms, with GPX1 and GPX4 being the most studied. However, in our study, only *gpx4* expression showed a significant increase due to the HE diet. This is consistent with published results, suggesting that GPX4 is the most important isoform in mammals and in fish, and is influenced by lipid levels [61,62]. Interestingly, GPX activity in the liver and white muscle showed the opposite pattern to that of its gene expression, presenting decreased levels in the HE groups. PRDX3 and PRDX5 are ubiquitous oxidoreductases that are mainly located in the mitochondria, which decrease hydrogen peroxide levels via their reduced thiol groups [63]. Furthermore, PRDX5 is involved in the regulation of adipogenesis by attenuating oxidative stress [60]. This is consistent with our results, which showed higher expression in the liver of the HE groups that also presented enlarged livers and altered mitochondrial biogenesis [18]. Similar interpretations were also made for the GPX-GR-GST and PRDX systems in the HE groups. They showed a general increase in their relative expression, but reduced enzyme activity, probably due to post-transcriptional regulation and limited NADPH availability. These effects were more pronounced in the liver but also in the white muscle. This reveals once again the importance of studying the redox status with different approaches [4]. Hence, to regulate the different peroxides, fish fed a high-lipid diet could prioritize the SOD-CAT pathway, while those fed a high-protein diet could use the GSH- and NADPH-related ones.

Oxidative stress also plays a key role in proteolysis by regulating protein degradation at different levels [64]. Chaperones (*calr*, *calnx*, and *hsp70*) are the tools associated with the quality control of proteins, being overexpressed to remove defective proteins via the ubiquitin–proteasome and cathepsin systems. In fact, we observed a significant increase in proteolytic systems in the white muscle of the VSHE group [19]. The increase in the relative expression of chaperones could be related to the AOPP levels [39,65]. Expression of the proapoptotic *casp3* gene was also increased in the VSHE group, but only in the liver, reinforcing the influence of dietary lipids on the redox status and cell integrity.

Overall, these results revealed that a commercial high-energy diet (HE) administered for 6 weeks increased the levels of biochemical redox markers, upregulated the expression of genes associated with oxidative stress, chaperones and proapoptotic-related processes at the systemic level, and modulated enzyme activity in fish performing voluntary swimming (VS), particularly in the liver. Nevertheless, the application of sustained aerobic physical activity (Ex) reduced many of these changes, producing values that matched those of the HP groups. These results could be relevant in the farming of gilthead sea bream up to market size.

### 4.2. Effect of Exercise on the Oxidative Stress Status

As detailed in the Introduction, moderate and sustained exercise (i.e., aerobic training) has multiple positive effects on fish performance. However, some unfavorable consequences have been reported. In the present study, we found significant differences in the biochemical redox markers in the different exercise groups, with AOPP and -SH levels being raised in the liver and the TBARS level being reduced in the white muscle of the Ex groups. The increase in the AOPP level suggests higher protein oxidation driven by the increased production of oxidant compounds and the boosted protein turnover due to exercise [20,66]. However, Zembron-Lacny et al. (2010) [67] showed reduced protein carbonyl levels after several weeks of training. Total -SH levels were increased in our Ex groups, suggesting that the training regimen improved the redox status of these fish. There are few studies on sustained aerobic exercise and its effect on thiols, since most are focused on the effect of a single high-intensity training regimen or on the use of nutritional supplements. In general, a training regimen applied for several weeks reduces some forms of reduced thiols in specific tissues [67,68,69]. However, contradictory results have also been reported [70,71]. Regarding lipid peroxidation, the reduced TBARS levels in the white muscle of the Ex groups was consistent with the findings of most studies. This could be associated with lean muscle due to a higher lipid mobilization and an increase in the activities of endogenous defenses such as GPX and GR [67,72,73]. Our results of the biomarkers analyzed suggest that the training regimen applied in this study improved the redox status of fish, especially in those on the high-fat diet.

The transcriptional regulation of the genes associated with oxidative stress was modulated by exercise in the different tissues. Sustained swimming generally increased the relative expression of these genes in the ExHP group and reduced it in the ExHE group when compared to their respective VSHP and VSHE groups. GST catalyzes the conjugation of GSH to electrophilic fragments from a wide range of substrates and has an important role in xenobiotic and lipid peroxide detoxification [74]. The relative expression of only *gst3* and *calr* in the liver were equally reduced in the Ex groups without any effect of the diet composition. However, the activity of the GST enzyme showed the opposite pattern in the same tissue. Once again, the lack of correlation between relative expression and activity suggests a post-transcriptional regulation of *gst3*. To our knowledge, there is no information about the relative expression of *gst3* in a sustained training trial, but our results on GST activity are consistent with previous findings in different tissues [70,75]. Regarding redox enzyme activities (SOD, CAT, GR, GPX and GST), it is generally accepted that they increase during sustained and moderate training. However, some contradictory results or interactions with other factors, such as diet, age and obesity, have been reported, contributing to the complexity in interpreting these variables [26,62,72,75,76,77,78]. We observed an interaction between the diet composition and exercise for SOD activity in the liver, with a small increase in the ExHP group and a reduction in the ExHE group when compared to the VSHP and VSHE groups, respectively. Camiletti-Moirón et al. (2014) [52] also found an interaction between dietary protein content and exercise for tSOD and SOD1 activities. However, in contrast to our results, they reported that the activities were increased with a normal protein diet but reduced by a high-protein diet. The combined effect of obesity and exercise was studied by Kapravelou et al. (2015) [62], who found interactions between the effects of phenotype (lean or obese) and exercise (sedentary or trained) on enzyme activities in the rat liver. SOD2 and CAT activities were reduced in the obese trained rats but increased in lean ones compared to their respective sedentary groups. On the other hand, SOD1 and GPX activities showed the opposite patterns in the same animals. In white muscle, the decrease in the relative expression of *sod1*, *cat*, *gpx4*, *prdx3* and *prdx5* alongside a lack of change in SOD, CAT and GPX activities and the improved levels of TBARS could indicate a negative transcriptional regulation of these enzymes once a redox equilibrium has been achieved. Pengam et al. (2020) [26] found similar results in the white muscle of rainbow trout subjected to moderate-intensity training such as that applied in this work. Kapravelou et al. (2015) [62] also showed different profiles for GPX activity and *gpx4* relative expression in the livers of rats subjected to training. Regarding the PRDX system, the significant downregulation in the relative expression of *prdx3* and *prdx5* in the white muscle and the suggested one for *prdx3* in the liver highlight the importance of this family of genes in exercise-induced signaling, as reviewed by Wadley et al. (2016) [63]. The expression of chaperones (*calr*, *calnx* and *hsp70*) was also modulated by training. However, the response depended on the diet provided, with a major effect found in the HE groups. The reductions in the ExHE group compared to the VSHE group suggest an improvement of the misfolded or damaged proteins [79]. The proapoptotic *casp3* gene involved in the cell proteolytic process is commonly associated with negative outcomes. However, the increase in *casp3* expression in white muscle could also be associated with muscle growth and reorganization through hypertrophy or hyperplasia in fish performing exercise, which is consistent with previously reported results [16,17,19]. Thus, sustained aerobic training had positive effects on the biochemical markers, downregulating the expression of several redox-related genes and modulating the activity of some enzymes in different tissues, especially in fish fed high-lipid diets.

## 5. Conclusions

The redox response in gilthead sea bream can be modulated by the diet composition and the swimming regimen, generating a systemic effect and altering biochemical marker levels, gene expression and enzyme activity. Furthermore, the physiological response can be affected by the interaction between these two factors. A high-lipid diet and sedentariness negatively impair the equilibrium between prooxidant compounds and antioxidant defenses in tissues, with a major impact in the liver. However, aerobic training ameliorates several of these redox markers, such as the relative expression of the genes associated with different pathways, the activities of the main enzymes, and the levels of biochemical compounds. Furthermore, different defense pathways might be activated by the presence of reducing agents such as NADPH and thiol groups originating from dietary protein. Sustained swimming partially equalized the physiological differences produced by the diets differing in their macronutrient proportions. Therefore, exercise emerges as a useful tool that allows the use of cheaper and more sustainable feedstocks, optimizing fish production without compromising the redox balance.

## Figures and Tables

**Table 1:** Composition of the high-protein (HP) and high-lipid (HE) diets.

	HP DIET	HE DIET
Digestible energy (MJ/kg)	18	19.9
Protein (% dry mass)	54	50
Lipids (% dry mass)	15	20
DHA (% dry mass)	1	1.4
EPA (% dry mass)	2.5	3
ARA (% dry mass)	0.2	0.4
DHA/EPA/ARA	5/12.5/1	3.5/7.5/1

DHA: docosahexaenoic acid; EPA: eicosapentaenoic acid; ARA: arachidonic acid; P: elemental phosphorus.

**Table 2:** Biochemical redox markers in the liver, white muscle and red muscle of gilthead sea bream fed a high-protein (HP) or high-lipid (HE) diet and performing voluntary swimming (VS) or sustained swimming (Ex) for 6 weeks.

		Voluntary Swimming		Exercise		ANOVA		
		High protein (VSHP)	High lipid (VSHE)	High protein (ExHP)	High lipid (ExHE)	*D*	*E*	*I*
	TBARS	0.65 ± 0.06	0.99 ± 0.09	0.68 ± 0.06	0.9 ± 0.1	***	-	-
**Liver**	AOPP	3.32 ± 0.43	5.03 ± 0.4	4.64 ± 0.4	5.67 ± 0.31	***	*	-
	-SH	262.5 ± 19.1	368.7 ± 17.2	336.8 ± 18.1	385.4 ± 20.8	***	*	-
	TBARS	0.43 ± 0.05	0.57 ± 0.06	0.37 ± 0.04	0.43 ± 0.04	*	*	-
**White muscle**	AOPP	6.04 ± 1.19	7.9 ± 2.01	4.58 ± 0.63	8.18 ± 0.85	*	-	-
	-SH	145.8 ± 3.7	137.8 ± 5.3	138.9 ± 5	132.7 ± 4.3	-	-	-
**Red muscle**	TBARS	0.99 ± 0.35	2.37 ± 0.72	0.81 ± 0.09	3.26 ± 0.82	**	-	-

Data are expressed as the mean ± SEM; TBARS, µmol MDA/mg protein; AOPP, µEq Ch-T/mg protein; and -SH, total reduced thiols presented as µmol/mg protein. Data for the background heat map were standardized against the mean value for each biochemical marker in each tissue. Purple and yellow indicate the lowest and highest concentration level, respectively, whereas white represents the 50th percentile. Two-way ANOVA with diet (D), exercise (E) and their interaction (I = D x E) as independent factors was performed for each marker: -, not significant; * *p* ≤ 0.05; ** *p* ≤ 0.01; and *** *p* ≤ 0.001.

## Data Availability

Data is contained within the article or Supplementary Material.

## Supplementary Materials

The following supporting information can be downloaded at: www.mdpi.com/article/10.3390/antiox11020319/s1. Table S1. Primers used for rt-qPCR: sequence, melting temperature and GenBank accession numbers.
