# Supplementary material for: Interaction between the Effects of Sustained Swimming Activity and Dietary Macronutrient Proportions on the Redox Status of Gilthead Sea Bream Juveniles (Sparus aurata L.)
Source: Antioxidants (Basel). 2022 Feb 6;11(2):319. doi: 10.3390/antiox11020319 (PMC8868478; doi:10.3390/antiox11020319)
Supplement: Supplementary file 1 [file antioxidants-11-00319-s001.zip › antioxidants-1580153-supplement-TableS1.pdf]

Table S1. Primers used for rt-qPCR: sequence, melting temperature and GenBank accession numbers

Type	Gene		Sequence 5′– 3′	Tm	Accession number
Housekeeping	*rps18*	F	GGGTGTTGGCAGACGTTAC	60	AM490061.1
		R	CTTCTGCCTGTTGAGGAACCA		
	rpl27a	F	AAGAGGAACACAACTCACTGCCCCAC	68	AY188520
		R	GCTTGCCTTTGCCCAGAACTTTGTAG		
	ef1a	F	CTTCAACGCTCAGGTCATCAT	60	AF184170
		R	GCACAGCGAAACGACCAAGGGGA		
	β-actin	F	TCCTGCGGAATCCATGAGA	60	X89920
		R	GACGTCGCACTTCATGATGCT		
Oxidative stress	*sod1*	F	CCATGGTAAGAATCATGGCGG	60	AJ937872
		R	CGTGGATCACCATGGTTCTG		
	sod2	F	CCTGACCTGACCTACGACTATGG	60	J0308833
		R	AGTGCCTCCTGATATTTCTCCTCTG		
	cat	F	TGGTCGAGAACTTGAAGGCTGTC	58	JQ308823
		R	AGGACGCAGAAATGGCAGAGG		
	gpx1	F	GAAGGTGGATGTGAATGGAAAAGATG	63	DQ524992
		R	CTGACGGGACTCCAAATGATGG		
	gpx4	F	TGCGTCTGATAGGGTCCACTGTC	61	AM977818
		R	GTCTGCCAGTCCTCTGTCGG		
	gst3	F	CCAGATGATCAGTACGTGAAGACCGTC	65	JQ308828
		R	CTGCTGATGTGAGGAATGTACCGTAAC		
	gr	F	CAAAGCGCAGTGTGATTGTGG	60	AJ937873
		R	CCACTCCGGAGTTTTGCATTTC		
	prdx3	F	ATCAACACCCCACGCAAGACTG	65	GQ252681
		R	ACCGTTTGGATCAATGAGGAACAGACC		
	prdx5	F	GAGCACGGAACAGATGGCAAGG	64	GQ252683
		R	TCCACATTGATCTTCTTCACGACTCC		
Chaperones and proapoptotic	*calr*	F	GGCGGCGGCTATGTGAAG	60	KF857313
		R	GCATCGCAGTCTGATCCAAGTC		
	calnx	F	CCCGAGGGTTGGCTAGATGA	60	KF857314
		R	GGCGTCTGGGTCTCCGATAT		
	hsp70	F	AATGTTCTGCGCATCATCAA	60	EU805481
		R	GCCTCCACCAAGATCAAAGA		
	casp3	F	CTGATCTGGATGGAGGCATT	60	EU722334
		R	AGTAGTAGCCTGGGGCTGTG		

F, forward; R, reverse; Ta, annealing temperature; *rpl27a*, ribosomal protein l27a; *rps18*, ribosomal protein s18; *ef1α*, elongation factor 1 alpha; *ß-act*, beta actin; *sod1*, superoxide dismutase 1; *sod2*, superoxide dismutase 2; *cat*, catalase; *gpx-1*, glutathione peroxidase 1; *gpx-4*, glutathione peroxidase 4; *gst3*, glutathione s-transferase 3; *gr*, glutathione reductase; *prdx3*, thioredoxin-dependent peroxide reductase 3; *prdx5*, thioredoxin-dependent peroxide reductase 5; *calr*, calreticulin; *calnx*, calnexin; *hsp70*, heat shock protein; *casp3*, caspase 3.
